# Healthcare utilization after stroke in Canada- a population based study

**DOI:** 10.1186/s12913-019-4020-6

**Published:** 2019-03-27

**Authors:** Adebimpe O. Obembe, Lisa A. Simpson, Brodie M. Sakakibara, Janice J. Eng

**Affiliations:** 1 Department of Physical Therapy, The University of British Columbia, Vancouver, Canada; 2 Rehabilitation Research Program, GF Strong Rehab Centre, Vancouver Coastal Health Research Institute, Vancouver, Canada; 3 Graduate Program in Rehabilitation Sciences, The University of British Columbia, Vancouver, Canada

**Keywords:** Stroke, Healthcare utilization, Health services, Mobility

## Abstract

**Background:**

More people are surviving stroke but are living with functional limitations that pose increasing demands on their families and the healthcare system. The aim of this study was to determine the extent to which stroke survivors use healthcare services on a population level compared to people without a stroke.

**Methods:**

This was a cross-sectional population-based survey that collected information related to health status, healthcare utilization and health determinants using the 2014 Canadian Community Health Survey. Healthcare utilization was assessed by a computer-assisted personal interview asking about visits to healthcare professionals in the last 12 months. Negative binomial regression was used to estimate the incidence rate ratios (IRR) and 95% confidence intervals (CI) for the number of health professional visits between stroke survivors and people without a stroke. The regression models were adjusted for demographics, as well as for mobility, mood/anxiety disorder and cardiometabolic comorbid conditions.

**Results:**

The study sample included 35,759 respondents (948 stroke, 34,811 non-stroke) and equate to 12,396,641 (286,783 stroke; 12,109,858 non-stroke) when sampling weights were applied. Stroke survivors visited their family doctor the most, and stroke was significantly associated with more visits to most healthcare professionals [e.g., family doctor IRR 1.6 (CI 1.4–1.8); nurse IRR 3.0 (CI 1.8–4.8); physiotherapist IRR 1.8 (CI 1.1–1.9); psychologist IRR 4.0 (CI 1.1–5.7)] except the dental practitioner, which was less [IRR 0.7 (CI 0.6–0.9)]. Mood/anxiety condition, but not cardiometabolic comorbid condition increased the probability of visiting a family doctor or social worker/ counsellor among people with stroke.

**Conclusion:**

Stroke survivors visited healthcare professionals more often than people without stroke, and were approximately twice as likely to visit with those who manage problems that may arise after a stroke (e.g., family doctor, nurse, psychologist, physiotherapist). The effects of a stroke include mobility impairment and mood/ anxiety disorders. Therefore, adequate access to stroke-related healthcare services should be provided for stroke survivors, as this may improve functional outcome and reduce future healthcare costs.

## Background

Stroke is a leading cause of long-term disability in adults worldwide [1] and it can be devastating to individuals. After age 55, there is a lifetime risk of stroke of 1 in 5 for women and 1 in 6 for men [2]. Stroke can result in loss of independence with immense human and financial burden, which will magnify as the world-wide incidence of stroke and stroke survivors continue to increase [3]. Donabedian [4] defined health utilization as the outcome of the interaction between health professionals and patients. Health utilization is a multidimensional process that includes indicators such as quality of care, accessibility, efficiency, equity, volume, continuity, comprehensiveness, productivity of care and healthcare expenses. [5]. Several factors (e.g. demographics, physical, psychological) might influence how stroke survivors use healthcare services [6–8]. In Canada, about 85 to 90% of stroke survivors return to their own environment with or without support services [9, 10]. While there are some data that suggest that stroke survivors may utilize greater healthcare services after their stroke [7, 8, 11, 12], studies have not compared this utilization using a control group. Impaired functional health, higher financial income, younger age, emotional distress [8], pre-stroke dependency [7], living arrangement, social circumstances [7] and access to a physician [6] have been associated with the use of healthcare services after stroke. Stroke survivors living at home have reported unmet needs in several domains, including mobility, mood, communication, health provision after discharge and managing stroke-related problems [13, 14]. These factors could plausibly affect healthcare utilization after stroke but their association has never been established.

Two-thirds of patients with a first stroke survive after 3 years, and the risk of surviving is lowest in the first year [15]. Pre-existing medical conditions, especially cardiometabolic conditions, are common among stroke patients, and can affect functional outcome [16]. Providing care for stroke survivors can be complex, requiring a continuum of coordinated health and support services which may include physicians and other allied health team members [17], although the utilization of these healthcare services has never been reported in a nationally representative population-based study of stroke survivors living in the community. It is widely recognized that healthcare systems lack continuity across services and are often criticized for shortening hospital length-of-stay and offering limited community services [18]. Therefore, the purpose of this study was to determine the effect of having a stroke on the annual visit rates to healthcare professionals using a population-based sample of community-dwelling adults in Canada. Given the multiple physical and cognitive impairments common in stroke, in addition to numerous pre-existing co-morbidities, we hypothesized that stroke survivors would utilize greater healthcare services compared to people without stroke.

## Methods

### Data source

Data were from the 2014 Canadian Community Health Survey– Annual Component (CCHS), a cross-sectional survey that collected information related to health status, healthcare utilization and health determinants for the Canadian population. Statistics Canada data did not require an ethics review as it is a secondary analysis, however, a proposal on the use of the data was approved by Statistics Canada. The CCHS covers the population 12 years of age and above, and living in private households in the ten provinces and the three territories, and it relies upon a large sample of respondents. Persons living on reserves and other Aboriginal settlements; full-time members of the Canadian Forces; institutionalized persons, children aged 12–17 years that are living in foster care, and persons living in the Quebec health regions of Région du Nunavik and Région des Terres-Cries-de-la-Baie-James, were excluded from the survey. On the whole, these exclusions represent less than 3 % of the Canadian population aged 12 years and older. Healthcare utilization was assessed by a computer-assisted personal interview asking about the number of visits to healthcare professionals [19]. Responding to the survey was voluntary. Respondents were asked whether they had conditions diagnosed by a healthcare professional that had lasted or was expected to last at least 6 months. Detailed descriptions of the survey are available elsewhere [19, 20].

### Inclusion and exclusion criteria

Our inclusion criteria required that individuals be at least 50 years of age at the time of the interview. Healthcare utilization rises slowly throughout adult life, and increases exponentially after the age of 50 years [21]. While the mean age of stroke is 69 years (and decreasing) [22], approximately 20% of stroke survivors are between the ages of 50 and 69 years [23].

### Variables

Respondents were stratified into two groups, stroke and non-stroke. The stroke group consisted of respondents who had suffered from the effects of a stroke that was expected to last or had already lasted 6 months or more [20]. The non-stroke group consisted of respondents who did not have a stroke and other major medical conditions (asthma, arthritis, cancer, chronic obstructive pulmonary disease, chemical sensitivity, scoliosis, back problem). Respondents were asked to provide information on age, sex, educational status, total household income and healthcare utilization. Respondents were asked how many times they had seen or talked to the following health professionals for care or advice about their physical, emotional or mental health in the last 12 months; family doctor or general practitioner, eye specialist, other medical specialists (e.g., allergist, orthopaedist, psychiatrist), nurse, dental practitioner (dentist/dental hygienist/orthodontist), chiropractor, physiotherapist, psychologist, social worker/counsellor, and audiologist/speech/occupational therapist. Respondents were stratified by sociodemographic variables (male/female; < or ≥ 65 years of age; < or ≥ $40,000 income) based on the literature. Individuals who are female, older and have a lower income have poorer outcomes after stroke [24, 25]. Respondents were also stratified into presence and absence of the following; mobility (able to walk with/without difficulty), mood or anxiety disorder (e.g., depression, bipolar disorder, mania, dysthymia, phobia, obsessive-compulsive disorder or a panic disorder), and cardiometabolic comorbid conditions (hypertension, diabetes, heart disease). These variables were selected because these are chronic medical conditions that are more likely to occur in stroke survivors [16, 26, 27] and it is well established that such physical and mental health impairments, as well as cardiometabolic comorbidities lead to poorer functioning [16] and quality of life [24, 25] after stroke.

### Statistical analysis

Descriptive statistics of weighted frequency, percent, mean and standard deviation were used to summarize the data. To account for survey design effects such as clustering and unequal selection probabilities, and to ensure that the results were representative of the Canadian population, the set of replicate sampling weights developed for the CCHS 2014 by Statistics Canada was used for all analyses. Negative binomial regression with incidence rate ratios (IRR) and 95% confidence intervals (CI) were used to investigate the association between variables. This technique is able to handle over-dispersed data as typically found with health provider visits [28]. Simple pair-wise associations (IRR and 95% CI) between the number of visits for each health professional and age, sex, education, income, mobility, mood/anxiety disorder and cardiometabolic comorbid condition explored factors associated with health utilization in the stroke group. Models were then built to investigate the relationship between dependent variables (visits to each healthcare professional) and the independent variable (group – stroke versus non-stroke) (Crude Model). Adjusted IRR and 95% CI were determined after controlling for sociodemographic covariates - age, sex, education, income (Model 1). Three additional models were explored by separately adding mobility (Model 1a), mood/anxiety disorder (Model 1b) or cardiometabolic comorbid condition (Model 1c) to control for physical and mental health conditions in stroke survivors.

Alpha level was set at 0.05. All analyses were performed with the IBM Statistical Package for Social Sciences (SPSS) for windows version 24.0 and Stata/ IC version 15 software package.

## Results

The study sample consisted of 35,759 respondents (948 stroke survivors and 34,811 non-stroke) across Canada and equate to an estimated 12,396,641 (286,783 stroke survivors; 12,109,858 non-stroke) when sampling weights were used. The mean age of the sample was 63.6 ± 10.0 years and 52.3% were women. The stroke group (mean = 70.5 ± 10.8 years) was older than the non-stroke group (mean = 63.4 ± 9.9 years). The stroke group had 1.5, 1.8 and 2.0 times the proportion of people with a mobility condition, cardiometabolic condition or mood/anxiety disorder, respectively compared to the non-stroke group (Table 1).

**Table 1:** Characteristics of study sample from the 2014 Canadian Community Health Survey

N (%)^a^	Total 12,396,641 (100)	Stroke 286,783 (2.3)	Non-stroke group 12,109,858 (97.7)
Age (Years) Mean (SD)	63.6 (10.0)	70.5 (10.8)	63.4 (9.9)
Age (Years) n(%)			
< 65	7,328,212 (59.1)	96,554 (33.7)	7,231,659 (59.7)
≥ 65	5,068,429 (40.9)	190,229 (66.3)	4,878,199 (40.3)
Sex n(%)			
Male	5,911,848 (47.7)	153,493 (53.5)	5,758,355 (47.6)
Female	6,484,793 (52.3)	133,290 (46.5)	6,351,503 (52.4)
Education n(%)			
< High School	1,455,086 (11.7)	66,868 (23.3)	1,388,218 (11.5)
≥ High School	10,941,555 (88.3)	219,915 (76.7)	10,721,639 (88.5)
Total Household Income (CAD) n(%)			
< 40,000	3,849,612 (31.1)	140,730 (49.1)	3,708,881 (30.6)
≥ 40,000	8,547,029 (68.9)	146,053 (50.9)	8,400,977 (69.4)
Mobility (Able to walk without difficulty) Yes n(%)	11,205,716 (90.4)	171,802 (59.9)	11,033,914 (91.1)
Had at least one cardiometabolic comorbid condition (Yes) n(%)	5,350,149 (43.2)	213,043 (74.3)	5,137,106 (42.4)
Had a mood/ anxiety disorder (Yes) n(%)	1,470,019 (11.9)	65,146 (22.7)	1,404,873 (11.6)

^a^Weighted distribution

The most common healthcare providers visited were the same between the stroke/non-stroke groups: family doctor (93.2%/ 84.7%), eye specialist (55.1%/ 50.2%), other medical specialists (54.0%/ 36.8%) and dental practitioner (43.9%/ 64.2%). The mean number of visits with the family doctor was 4.9 visits/ person/ year [Standard Deviation (SD) =5.9] for the stroke group, and 3 visits/ person/ year (SD =4.8) for the non-stroke group. Proportion and mean number of visits to healthcare providers are presented in Table 2.

**Table 2:** Mean number of health professional visits reported by samples in the 2014 Canadian Community Health Survey

Variable	Total		Stroke		Non-Stroke group	
Visited Healthcare Provider (Yes)	N (%)	Mean (SD)	n (%)	Mean (SD)	n (%)	Mean (SD)
Family Doctor	10,521,679 (84.9)	3.0 (4.8)	267,142 (93.2)	4.9 (5.9)	10,254,536 (84.7)	3.0 (4.8)
Eye Specialist	6,235,330 (50.3)	1.1 (5.8)	157,994 (55.1)	1.4 (6.9)	6,077,336 (50.2)	1.1 (5.8)
Other Medical Specialists	4,611,415 (37.2)	1.1 (3.6)	154,815 (54.0)	2.2 (10.3)	4,456,601 (36.8)	1.1 (3.2)
Nurse	1,503,285 (12.1)	0.9 (10.4)	79,446 (27.7)	3.2 (17.6)	1,423,838 (11.8)	0.9 (10.2)
Dental Practitioner	7,901,339 (63.7)	1.3 (1.6)	125,892 (43.9)	1.0 (1.8)	7,775,447 (64.2)	1.3 (1.6)
Chiropractor	1,485,209 (12.0)	1.1 (5.1)	23,912 (8.3)	1.1 (7.7)	1,461,297 (12.1)	1.1 (5.0)
Physiotherapist	1,588,690 (12.8)	1.3 (6.9)	46,142 (16.1)	1.9 (8.1)	1,542,548 (12.7)	1.2 (6.9)
Psychologist	272,087 (2.2)	0.2 (1.8)	8792 (3.1)	0.4 (4.2)	263,295 (2.2)	0.2 (1.7)
Social Worker/ Counsellor	429,440 (3.5)	0.2 (2.8)	29,412 (10.3)	0.7 (6.4)	400,028 (3.3)	0.2 (2.1)
Audiologist/ Speech or Occupational Therapist	653,850 (5.3)	0.1 (1.2)	34,038 (11.9)	0.4 (1.9)	619,812 (5.1)	0.1 (1.1)

N (%) – Visited healthcare provider at least once during the last 12 months

*SD* Standard deviation

From the simple pair-wise associations in the stroke group (Table 3), higher income was related to visits to dental practitioners and psychologists (IRR 1.5–8.8), and mood/anxiety disorder was related to visits to family doctors, other medical specialists, psychologists and social worker/counsellors (IRR 1.4–22.1). Age (≥ 65 years), male sex and cardiometabolic comorbid condition was related to visits to eye specialists (IRR 1.7–1.9). Stroke survivors who were able to walk without difficulty (mobility) visited the eye specialists and social worker/counsellors less (IRR 0.2–0.5).

**Table 3:** Pair-wise associations of healthcare utilization of stroke survivors

Variable IRR (95% CI)	Family Doctor	Eye Specialist	Other Medical Specialists	Nurse	Dental Practitioner	Chiropractor	Physiotherapist	Psychologist	Social Worker/ Counsellor	Audiologist/ Speech or Occupational Therapist
Age (Years)										
< 65^c^										
≥ 65	0.8 (0.6–1.1)	1.7 (1.0–2.9)^a^	0.4 (0.2–0.7)^b^	1.4 (0.5–3.7)	1.7 (0.4–1.2)	0.4 (0.1–4.3)	0.6 (0.2–1.5)	0.1 (0.0–1.9)	0.4 (0.1–1.4)	0.5 (0.2–1.3)
Sex										
Female^c^										
Male	0.9 (0.6–1.2)	1.7 (1.0–2.7)^a^	0.7 (0.5–1.1)	1.2 (0.5–3.1)	1.1 (0.8–1.7)	0.3 (0.1–1.1)	1.1 (0.4–2.8)	1.9 (0.4–10.0)	2.3 (0.7–7.7)	0.9 (0.4–1.7)
Highest level of Education										
< High School^c^										
≥ High School	1.1 (0.8–1.3)	1.1 (0.6–1.9)	1.0 (0.5–1.8)	0.6 (0.2–1.5)	1.0 (0.5–1.8)	2.0 (0.4–10.3)	0.9 (0.1–7.1)	0.2 (0.0–7.2)	1.5 (0.4–5.3)	1.5 (0.5–4.4)
Income (CAD)										
< $40,000^c^										
≥ $40,000	1.0 (0.7–1.3)	0.6 (0.3–1.1)	0.9 (0.6–1.4)	0.7 (0.2–1.8)	1.5 (1.0–2.2)^a^	2.1 (0.4–9.6)	1.3 (0.5–3.3)	8.8 (1.6–49.1)^a^	0.7 (0.2–2.7)	1.3 (0.6–3.0)
Mobility										
Cannot walk^c^										
Can walk	0.8 (0.6–1.1)	0.5 (0.3–0.9)^b^	0.8 (0.5–1.3)	0.6 (0.2–1.5)	1.1 (0.7–1.7)	0.5 (0.1–1.9)	1.2 (0.4–3.3)	0.7 (0.1–3.8)	0.2 (0.1–0.6)^b^	0.5 (0.2–1.1)
Mood/ Anxiety										
No^c^										
Yes	1.4 (1.1–1.8)^a^	1.5 (0.7–3.0)	1.8 (1.0–3.1)^a^	2.4 (0.8–6.8)	1.1 (0.7–1.9)	1.4 (0.3–5.5)	1.0 (0.2–5.2)	22.1 (3.2–149.8)^a^	7.4 (2.5–21.9)^a^	2.1 (0.8–5.1)
Cardiometabolic Comorbid Condition										
No^c^										
Yes	1.1 (0.8–1.5)	1.9 (1.2–2.8)^a^	1.1 (0.6–2.2)	1.9 (0.7–5.0)	0.9 (0.6–1.4)	0.3 (0.1–2.2)	0.4 (0.1–1.8)	1.6 (0.1–45.3)	0.4 (0.1–3.6)	1.5 (0.6–4.3)

*IRR (95% CI)* Incidence rate ratio (95% confidence interval)

^a^Significantly associated with higher healthcare service visits after stroke

^b^Significantly associated with lower healthcare service visits odds after stroke

^c^Reference category

Table 4 shows a summary of IRRs for annual visits to healthcare professionals, comparing stroke and non-stroke groups in the regression models. When unadjusted (Crude Model), stroke survivors visited the family doctor, other medical specialists, nurse, psychologist, social worker/counsellor and audiologist/ speech or occupational therapist significantly more (IRR 1.6–3.7) and visited dental professionals less than people without stroke (IRR 0.7). The stroke survivors had higher visitation rates to these healthcare professionals (IRR 1.6–3.2) than the non-stroke group. After controlling for sociodemographic covariates (age, sex, education and income), the health professions that showed greater visits by stroke survivors in the Crude Model continued to be significant (IRR 1.6–3.2), in addition to visits to physiotherapists and psychologists (Model 1). The models also showed that stroke survivors have higher visitation rates to these healthcare professionals after mobility (IRR 1.3–3.0), mood/anxiety disorder (IRR 1.5–3.3) and cardiometabolic comorbid condition (IRR 1.4–5.1) were controlled (Models 1a - 1c).

**Table 4:** Multi-level negative binomial regression models showing the comparison of health professional visits between stroke and non-stroke groups

Variable	Crude Model IRR (95%CI)	Model 1 IRR (95%CI)	Model 1a IRR (95%CI)	Model 1b IRR (95%CI)	Model 1c IRR (95%CI)
Family Doctor	1.6 (1.4–1.9)^a^	1.6 (1.4–1.8)^a^	1.3 (1.1–1.5)^a^	1.5 (1.3–1.7)^a^	1.4 (1.2–1.7)^a^
Eye Specialist	1.2 (0.9–1.7)	1.1 (0.8–1.6)	1.0 (0.8–1.4)	1.1 (0.8–1.5)	1.1 (0.8–1.4)
Other Medical Specialists	2.0 (1.4–2.9)^a^	2.0 (1.4–2.8)^a^	1.7 (1.2–2.4)^a^	1.9 (1.3–2.6)^a^	1.9 (1.3–2.6)^a^
Nurse	3.7 (2.3–6.1)^a^	3.0 (1.8–4.8)^a^	2.7 (1.4–5.2)^a^	2.6 (1.6–4.2)^a^	2.6 (1.6–4.1)^a^
Dental Practitioner	0.7 (0.6–0.9)^b^	0.9 (0.7–1.1)	0.9 (0.8–1.2)	0.9 (0.7–1.1)	0.9 (0.7–1.1)
Chiropractor	1.0 (0.4–2.5)	1.3 (0.5–2.5)	1.2 (0.5–2.6)	1.2 (0.5–2.9)	1.2 (0.5–2.9)
Physiotherapist	1.5 (0.9–2.5)	1.8 (1.1–1.9)^a^	1.8 (1.1–2.9)^a^	1.8 (1.2–2.9)^a^	1.9 (1.1–3.1)^a^
Psychologist	2.9 (0.3–29.8)	4.0 (1.1–5.7)^a^	4.5 (0.7–30.1)^a^	6.2 (0.4–90.1)^a^	4.1 (0.7–22.5)
Social Worker/ Counsellor	3.1 (1.7–5.6)^a^	3.2 (2.2–4.8)^a^	3.0 (1.5–5.8)^a^	3.3 (1.6–6.9)^a^	5.1 (1.9–13.1)^a^
Audiologist/ Speech or Occupational Therapist	3.2 (1.9–5.4)^a^	2.9 (1.2–2.1)^a^	2.5 (1.3–5.1)^a^	2.9 (1.6–5.3)^a^	2.9 (1.7–5.1)^a^

Crude Model - Unadjusted incidence rate ratios

Model 1 - Incidence rate ratios were adjusted for age, sex, education and income

Model 1a - Incidence rate ratios were adjusted for age, sex, education, income and mobility

Model 1b - Incidence rate ratios were adjusted for age, sex, education, income and mood/anxiety disorder

Model 1c - Incidence rate ratios were adjusted for age, sex, education, income and presence of cardiometabolic comorbid condition

*IRR (95% CI)* Incidence rate ratio (95% confidence interval)

^a^Stroke survivors significantly associated with higher healthcare service visits than those without stroke

^b^Stroke survivors significantly associated with lower healthcare service visits than those without stroke

## Discussion

The burden that stroke constitutes for patients, their families and the healthcare system is substantial [29]. Corresponding to our hypothesis, stroke survivors were more likely to visit most healthcare professionals than those without stroke using a large population-based sample with a comparison control group without stroke, and this suggests that community-dwelling individuals with stroke need more care and may have greater health needs because of their health condition. Stroke survivors visited the family doctor more than any of the other health professionals. The stroke sample in our study was more likely to visit healthcare professionals that typically manage problems that may arise after a stroke (e.g., family doctor, nurse, physiotherapist, audiologist/speech therapist, occupational therapist, psychologist).

Even after the models were adjusted for sociodemographic variables and the presence of a mobility, mood/anxiety or cardiometabolic condition, the greater number of health professional visits still remained for the stroke group. It is possible that the severity of these conditions may have influenced the number of visits as only the presence/absence of these conditions was considered, and not a finer gradation such as walking speed for mobility or actual resting blood pressure for cardiometabolic risk. Certainly the mobility impairments of stroke survivors can be complex and severe with partial muscle paralysis, sensory loss, spasticity and ataxia.

There was a much higher prevalence of a mobility impairment, mood/anxiety disorder or cardiometabolic condition in the stroke group compared to the control, and consequently a higher prevalence of having multiple conditions which may require visits to the family doctor. Thus, it is possible that interactions among these conditions may have had impacted the results, for example, mobility problems may contribute to anxiety, while low mood may deter one from activities that keep one mobile. Interestingly, stroke survivors with walking difficulties visited a social worker/counsellor around five times more often than stroke survivors without a walking difficulty. A number of explanations may account for this finding, including the evidence that those with walking difficulties have more psychosocial difficulties [30] or the role of social workers in organizing mobility and transportation services to address physical impairments [31]. In addition, other mediators may be involved as mobility ability has been shown to relate to overall stroke severity [32] and social workers/counsellors likely prioritize individuals who are more impacted by their stroke.

The greatest discrepancy in visits was with psychologists where stroke survivors visited four to six times that of people without stroke. Stroke survivors with mood/anxiety disorders were far more likely to visit a psychologist, but not their family doctor than stroke survivors without a mood/anxiety disorder. Furthermore, while 22.7 and 11.6% of the stroke and non-stroke groups reported a mood/anxiety disorder, the proportions visiting a psychologist were very small (2–3% in both groups). Stigmas, as well as a lack of knowledge about mental health symptoms and treatments may prevent people from seeking treatment for depression or anxiety; a US national study of 21,000 males showed that less than 50% sought treatment, despite experiencing depression or anxiety on a daily basis [33]. Psychological disorders have also been linked to greater dependence in activities of daily living, poorer quality of life after stroke [34] and higher utilisation of healthcare services if untreated [35–37]. A barrier to most community-based psychological services is the fact that they are delivered by private services, making it unaffordable for many people [38]. Also, psychological services are not covered by health insurance, making it even more unaffordable. Sixty-five percent of Canadians have private health insurance which pays for the health-care expenditures that go towards other professionals (dentists, optometrists and physiotherapists, among others) [39]. Private health insurance has been suggested to be a predictor of improved outcomes after stroke [40].

The findings from this study have considerable importance and implications as this study was based on a large sample of adults with or without stroke in Canada. The results suggest that stroke survivors have stroke-related health needs that requires more visits to healthcare professionals than people without stroke. Stroke survivors with disabilities usually require care that can be complex due to their multiple needs. These individuals might need to consult several healthcare professionals for different medical conditions. Their complex care requires easy accessibility to healthcare services professionals. It is essential for the healthcare system to be responsive to these needs. Interdisciplinary health services delivery programs, involving healthcare professionals crucial to stroke management, should be developed for the care of these patients.

There are several limitations that warrant acknowledgment in this study. Firstly, stroke and other conditions were self-reported by individuals and not verified by any other source. Self-report measures are easily implemented to large samples, but have limitations such as recall/ response bias, introspective ability and social desirability bias. The study involved only people living in private households, therefore, the results may not be generalized to all stroke survivors as there are some who are residents of healthcare institutions. As administrative data were used, many variables that were potential cofounders of stroke were not assessed or provided, including clinical parameters (such as stroke type, stroke severity), family structure and support network, as well as use of other healthcare resources, such as ambulance use and emergency room admissions. Therefore, not all probable cofounding factors of stroke were adjusted for because the details were not available in this study. We adjusted for mobility and comorbidities, but we were not able to adjust for other determinants of stroke outcomes such as stroke type and severity (however, mobility can be considered one surrogate for severity). Further studies that will include more detailed stroke risk factors and comorbidities are needed to address these limitations.

## Conclusion

Stroke survivors visited healthcare professionals more than people without stroke, and were more likely to visit those that manage problems that may arise after a stroke (e.g., family doctor, nurse, psychologist, physiotherapist). The effects of a stroke include mobility impairment and mood/ anxiety disorders. Therefore, adequate access to stroke-related healthcare services should be provided for stroke survivors, as this may improve functional outcome and reduce future healthcare costs.

## Abbreviations

CFI: Canadian Foundation for Innovation
CI: 95% confidence intervals
CIHR: Canadian Community Health Survey
CIHR: Canadian Institutes of Health Research
CRDCN: Canadian Research Data Centre Network
IRR: Incidence rate ratios
SD: Standard deviation
SSHRC: Social Sciences and Humanities Research Council
